# STAS More than a Prognostic Marker—An Evolving Factor in Operative and Adjuvant Treatment Decisions in Early-Stage NSCLC

**DOI:** 10.3390/cancers18091414

**Published:** 2026-04-29

**Authors:** Joshua R. Brady, Andrea L. Axtell

**Affiliations:** 1 Department of Surgery, University of Wisconsin Hospitals and Clinics, Madison, WI 53792, USA; jbrady@uwhealth.org; 2 Division of Cardiothoracic Surgery, Department of Surgery, University of Wisconsin School of Medicine and Public Health, Madison, WI 53792, USA

**Keywords:** spread through air spaces, STAS, early-stage NSCLC, sublobar resection

## Abstract

Since tumor spread through air spaces (STAS) was first described over a decade ago, numerous studies have demonstrated that it is a high-risk prognostic feature in non-small cell lung cancer (NSCLC). However, due to preoperative and intraoperative limitations in pathologic diagnosis, STAS is generally diagnosed following curative intent resection. While STAS should influence NSCLC treatment strategy—particularly upfront surgical decision-making—postoperative diagnosis of STAS has heretofore limited this possibility. While limited to retrospective studies, the current evidence suggests that patients with tumor STAS should undergo a more extensive anatomical resection—preferably a lobectomy, if they are candidates. These results are particularly important in the setting of the results of the JCOG0802 and CALGB 140503 randomized controlled trials which have begun a paradigm-shift toward sublobar resections for early-stage NSCLC, which may not hold similar benefit for early-stage STAS+ disease. The aims of this review are to: (1) detail the current evidence concerning choice of resection extent for STAS+ disease, (2) summarize the current evidence about optimum surgical margins for STAS+ disease, (3) detail the potential role for adjuvant chemotherapy in early-stage STAS+ disease, (4) assess the current limitations in preoperative STAS risk prediction and intraoperative STAS detection, and (5) highlight promising AI-based advancements which will allow surgeons to risk-stratify STAS probability or confirm STAS status intraoperatively. The main limitation of this review is the reliance on retrospective studies as there is a current lack of prospective or randomized data within STAS+ NSCLC, particularly regarding optimal resection strategy for STAS+ disease.

## Simple Summary

Despite a growing body of evidence demonstrating that tumor spread through air spaces (STAS) is a high-risk prognostic feature in non-small cell lung cancer (NSCLC), the integration of STAS into NSCLC treatment algorithms continues to be inconsistent without universal agreement on how STAS should influence operative and adjuvant treatment choices. This review demonstrates that STAS should be viewed as a pathologic feature which directly influences operative and adjuvant therapy choices.

## 1. Introduction

Lung cancer remains the leading cause of cancer-related death worldwide with an estimated 1.8 million deaths worldwide in 2020, and 130,000 deaths annually in the United States [1,2]. Despite improvements in early detection and treatments, non-small cell lung cancer (NSCLC), which accounts for 80–85% of lung cancer diagnoses, continues to have a poor five-year survival of approximately 25–30% [3,4]. For early-stage NSCLC, surgical resection remains the standard of care with recent randomized controlled trials (RCTs: CALGB 14503 and JCOG0802) suggesting that a sublobar resection (defined as either a segmentectomy or a wedge resection) may be a superior or noninferior resection compared to a lobectomy for stage IA disease less than or equal to 2 cm [5,6]. Despite undergoing surgical resection, recurrence rates remain high in early-stage NSCLC [7]. Furthermore, the five-year relative survival rate for early-stage NSCLC is approximately 67%, with considerable room for improvement [2].

In light of these RCTs results and current NSCLC outcomes, there is an increasing focus on the importance of prognostic biomarkers and histopathologic features in guiding therapy. Biomarkers, such as EGFR, ALK, and PD-L1, have become important not only for prognosis and monitoring, but also for the development of effective targeted therapies and immunotherapies [8,9]. However, only an estimated 60% of NSCLC tumors have biomarkers which are targetable with therapy [10]. There are additional significant limitations within prognostic biomarkers including spatial and temporal tumor heterogeneity and variation in detection methods across testing methods and specimens [9]. Furthermore, while rapid advancements have been made regarding the development of diagnostic and prognostic NSCLC biomarker panels, ongoing optimization and validation continues to be required [9,11]. Given the continued poor survival rate for NSCLC and that 40% of tumors lack actionable biomarkers, prognostic biomarkers must be viewed in conjunction with other disease features which can characterize disease aggressiveness such as histopathologic features.

Histopathologic features, including visceral pleural invasion (VPI), which automatically upstages tumors to at least T2a, and lymphovascular invasion (LVI), have been demonstrated to be strong independent prognostic factors for poor outcomes, particularly in early-stage NSCLC [12,13,14,15]. More recently, tumor spread through air spaces (STAS) was recognized as an important histopathologic feature with increasing evidence that it is also a poor prognostic factor in NSCLC [16,17]. Due to the clinical importance of STAS, it was recently included as a histologic descriptor in the Ninth Edition of the TNM Classification of lung Cancer released in January 2025, but it does not currently change the official T staging [18].

Given the increasing evidence that STAS is an independent negative prognostic factor in NSCLC, STAS should influence operative and adjuvant treatment choices. However, integration of STAS into NSCLC treatment algorithms remains inconsistent and limited due to limitations in the diagnosis of STAS. Despite advancements in preoperative and intraoperative detection of STAS, histopathological examination remains the gold standard for confirming STAS [19,20]. In this review we will detail the latest advancements in STAS detection and the impact of STAS on NSCLC outcomes to reframe how STAS should be viewed as a pathologic feature which directly influences operative and adjuvant therapy choices. We will also detail current limitations within STAS detection and areas requiring prospective or RCTs.

## 2. STAS: Definition and Pathologic Characteristics

### 2.1. Formal Definition of STAS

While first documented in 1980 by Kodama et al., STAS was not formally classified until 2015 [21]. In 2015, the World Health Organization (WHO) first included the concept of STAS in the Classification of Lung Tumors, defining it as “spread of micropapillary clusters, solid nests, and/or single cancer cells into airspaces in the lung parenchyma beyond the edge of the main tumor” [22,23].

### 2.2. Morphologic Patterns of STAS

**Figure 1:**
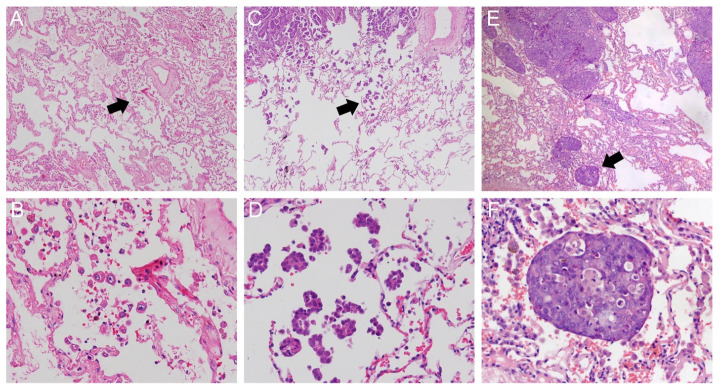
Morphologic Patterns of STAS. Single cell pattern (black arrow) under ×40 magnification (**A**) and ×200 magnification (**B**). Micropapillary cluster pattern (black arrow) under ×40 magnification (**C**) and ×200 magnification (**D**). Solid nest pattern (black arrow) under ×40 magnification (**E**) and ×200 magnification (**F**). Reproduced via Creative Commons CC BY license without alteration from: Xie H, Su H, Zhu E et al. Morphological Subtypes of Tumor Spread Through Air Spaces in Non-Small Cell Lung Cancer: Prognostic Heterogeneity and Its Underlying Mechanism. *Front Oncol*. 2021;11:608353. Doi: 10.3389/fonc.2021.608353 [25].

The three morphologic patterns of STAS (micropapillary clusters, solid nests, and single cells) were first described by Kadota et al. [24]. Examples of all three morphologic patterns in pathologic specimens are shown in Figure 1 [25]. Across all NSCLC, micropapillary clusters are the most prevalent of the three morphologic patterns with studies demonstrating incidences ranging from 58.2% to 80% [26,27]. All three morphologic patterns have been exhibited in lung adenocarcinoma with micropapillary clusters being the most common across all histologic subtypes [24,26,28]. Conversely, the solid nests morphologic pattern is the only pattern exhibited in lung squamous cell carcinoma [29,30,31]. These different morphologic patterns may represent different underlying molecular alterations with a potential association between ALK rearrangements and micropapillary clusters [28].

### 2.3. Distinction Between True STAS and Artifact

When first defining STAS, it was acknowledged that tumor processing artifacts could potentially mimic STAS [24]. In fact, the original description of STAS by Kadota et al. included specific guidelines to assist in distinguishing STAS from tissue-processing artifacts (e.g., jagged edges or detached tissue) [24,32]. Despite this, it has been questioned whether STAS was a true in vivo phenomenon or potentially an ex vivo artifact from processing [33,34]. However, growing evidence has demonstrated the presence of STAS and its association with poor prognosis in NSCLC, advancing the notion that it is an in vivo phenomenon.

Studies that examined the reproducibility of distinguishing artifact from STAS demonstrated good reproducibility. For example, a study by Baine et al. of 30 images reviewed by 10 pathologists from 5 institutions demonstrated unanimous agreement in 80% of cases and an average kappa value of 0.857 (range: 0.614–1.00) [32,35]. In addition, Metovic et al., in a prospective cohort of 51 surgical lung specimens, demonstrated no significant difference in STAS occurrence or STAS distribution based on gross specimen handling procedures [36]. Furthermore, a retrospective analysis of pathologic specimens from 10 STAS+ NSCLC patients’ post-sublobar resection who had also undergone additional resection (completion lobectomy or additional sublobar (wedge resection or segmentectomy resection)) by Gross et al. evaluated the presence of STAS in both the initial surgical resection and the additional resection specimen using different processing knives at different times [17]. Despite all patients undergoing R0 sublobar resections, STAS was detected in the additional resection specimens in 100% of patients [17]. This is additional strong evidence against STAS representing an artifact from tissue processing [17]. Together, these studies support STAS being an in vivo invasive mechanism similar to lymphovascular or pleural invasion.

## 3. STAS Epidemiology and Histologic Associations

### 3.1. Frequency of STAS in NSCLC

In retrospective studies, STAS is reported in approximately 20–50% of resected NSCLC specimens depending on the study population, tumor stage, and histology [16,28,37,38]. A recent meta-analysis of 47 studies, spanning all NSCLC types and tumor stages estimated the overall rate of STAS in NSCLC patients to be 0.368 (95% CI: 0.336–0.401) [39].

### 3.2. STAS Histologic Associations

Tumor STAS is significantly associated with the adenocarcinoma histologic type of NSCLC [40]. Despite the strong association with adenocarcinoma, STAS has also been noted in other NSCLC histologic types including squamous cell carcinoma and pleomorphic carcinoma as well as other lung malignancies including neuroendocrine tumors (i.e., typical carcinoid, atypical carcinoid, large cell neuroendocrine carcinoma, and small cell lung cancer) [30,41,42]. A recent meta-analysis estimated the rate of STAS in adenocarcinoma to be 0.374 (95% CI: 0.340–0.409) and in squamous cell carcinoma to be 0.338 (95% CI: 0.273–0.411) [39].

### 3.3. Adenocarcinoma Histologic Subtypes and Tumor STAS

While adenocarcinoma is most commonly associated with STAS, not all adenocarcinoma histologic subtypes demonstrate equal presence of STAS. As first noted by Kadota et al., STAS is more frequently associated with micropapillary, papillary, and solid histologic subtypes and less commonly associated with the lepidic histologic subtype [24]. The meta-analysis by Pyo et al. also demonstrated that, among adenocarcinoma histologic subtypes, micropapillary-predominant tumors exhibited the highest STAS prevalence (0.719, 95% CI: 0.652–0.778) [39]. In addition to micropapillary tumors, the solid (0.567, 95% CI: 0.478–0.652) and papillary (0.446, 95% CI: 0.392–0.501) subtypes demonstrated significant STAS occurrence [39]. Conversely, lepidic-predominant tumors demonstrated the lowest prevalence of STAS of the subtypes (0.128, 95% CI: 0.092–0.175) [39].

## 4. Prognostic Implications of STAS

While current investigations have been limited to retrospective cohort studies, there is growing evidence that tumor STAS has prognostic implications for both overall survival and tumor recurrence.

### 4.1. Association Between STAS and Recurrence

The association between STAS and increased recurrence has been suggested by several studies. It was demonstrated early in the identification of STAS as a pathologic phenomenon. In a retrospective study of 411 stage I ≤ 2 cm adenocarcinoma patients, the risk of recurrence was significantly increased in STAS+ tumors following sublobar resection (5-year cumulative incidence of recurrence, 42.6% versus 10.9%; *p* < 0.001) with significantly increased risk of both distant (*p* = 0.035) and locoregional recurrence (*p* = 0.001) [24]. STAS remained an independent risk factor for recurrence in multivariate analysis [24]. Additionally, a retrospective study of 809 stage IA lung adenocarcinoma patients by Vaghjani et al. revealed a significant association between STAS and occult lymph node metastasis on both univariate (67% vs. 39%, *p* < 0.001) and multivariable analysis (*p* = 0.004) [43]. Retrospective studies have also demonstrated a significant association between STAS and the development of distant metastases with a study of 207 stage I ≤ 2 cm adenocarcinoma patients by Buzás et al. showing a significant association between STAS and brain metastases (19.2% vs. 7.7%, *p* = 0.019) [44].

### 4.2. Associations Between STAS and Recurrence-Free and Overall Survival

Numerous retrospective studies have demonstrated negative associations between the presence of STAS and both recurrence-free survival (RFS) and overall survival (OS). A retrospective study of 318 stage I lung adenocarcinoma patients demonstrated that compared to STAS- patients, STAS+ patients had on univariate analysis significantly decreased five-year OS (62.7% vs. 91.1%, *p* < 0.01), and decreased five-year RFS (54.4% vs. 87.8%, *p* < 0.01), which remained significant on multivariate analysis [45]. A recent meta-analysis showed that STAS was significantly associated with decreased OS (HR: 2.119, 95% CI: 1.811–2.480) and RFS (HR: 2.372, 95% CI: 2.018–2.788) [39].

Moreover, studies that graded STAS (either semi-quantitatively or via distance from tumor edge) have demonstrated that advanced STAS grade led to worse patient survival. In a retrospective study of 1869 NSCLC cases, Han et al. graded STAS by its extent (STAS I: <2500 μm [one field of ×10 objective lens] from the edge of tumor and STAS II: ≥2500 μm from the edge of tumor) and noted a significant inverse relationship between STAS extent and both RFS and OS [16]. Furthermore, a retrospective study of 73 lung adenocarcinoma patients by Gutierrez-Sainz et al. demonstrated a significant association between the distance from tumor edge to farthest STAS with a ≥1.5 mm distance having a significantly reduced median RFS (37.63 months, 95% CI: 28.14–47.11 months) and every 1 mm increase in distance increase increasing the mortality risk by 1.26 times (*p* = 0.04) [46]. Uruga et al. retrospectively evaluated the presence of STAS in 208 stage I ≤ 2 cm adenocarcinomas and semi-quantitatively classified patients as STAS-, low STAS (1–4 single cells or clusters of STAS), or high STAS (≥5 single cells or clusters of STAS) [47]. They found that increasing STAS was significantly associated with decreased RFS on univariate analysis and on multivariate Cox proportional hazards analysis (*p* = 0.015) [47].

### 4.3. Prognosis in Late-Stage NSCLC

While studies investigating the prognostic impact of STAS have predominately focused on early-stage lung adenocarcinoma, retrospective studies also suggest that STAS is also associated with worse prognosis in late-stage NSCLC. Warth et al. noted that STAS was associated with both significantly decreased OS (*p* = 0.020) and disease-free survival (DFS, *p* = 0.004) in a retrospective study of 569 resected lung adenocarcinomas of all stages [48]. Of note, they found increased prevalence of STAS in high stage (*p* < 0.001), node positive (*p* < 0.001) adenocarcinomas with distant metastases (*p* = 0.010) [48]. Additionally, a retrospective study of 76 stage III (N2) lung adenocarcinoma patients by Terada et al. demonstrated that STAS was significantly correlated with recurrence risk on univariate analysis (HR: 1.95, 95% CI: 1.07–3.51, *p* = 0.029) [49].

## 5. Impact of STAS on Operative Decision-Making

While recent RCTs have demonstrated that sublobar resection (defined as a segmentectomy or wedge resection) is noninferior to lobectomy in unselected stage I (≤2 cm) NSCLC, the optimal surgical resection for NSCLC with STAS remains unclear [5,6]. In the sections below, sublobar resection will include both wedge resection and segmentectomy.

### 5.1. Comparing Outcomes in STAS+ Patients Following Sublobar vs. Lobar Resections

The most important operative choice is the extent of resection. To determine the optimal operation for STAS, several retrospective studies have been performed, mainly comparing outcomes in sublobar resections (wedge resection or segmentectomy) to lobar resections (aka lobectomy) in stage I tumors. These studies are detailed in Table 1. Currently, investigations into outcomes of STAS+ patients following sublobar and lobar resections are limited to retrospective studies with evidence from prospective or RCTs. Most of these retrospective studies showed that in STAS+ tumors, a sublobar resection was associated with worse recurrence and survival [24,38,50,51,52,53,54,55,56,57]. A meta-analysis of 11 studies (5097 patients) in stage I lung adenocarcinoma by Yang et al. redemonstrated that in STAS+ patients, sublobar resection resulted in worse RFS and OS than a lobectomy [58]. Furthermore, more extensive STAS (increased tumor cell clusters and/or single tumor cells) in sublobar patients was associated with significantly worse RFS and OS [56].

Limited studies have separated the two different types of sublobar resection (segmentectomy and wedge resection) in their analysis and directly compared wedge resection to lobectomy and/or segmentectomy to lobectomy. The results of the few studies which have differentiated between wedge resection and segmentectomy potentially suggest different outcomes within sublobar resections in STAS+ disease. This is an important distinction as a wedge resection is not an anatomical resection (like a segmentectomy or lobectomy) [51,59,60]. For example, a retrospective study of 609 (293 STAS+) stage IA lung adenocarcinoma patients by Kagimoto et al. revealed, among STAS+ patients, no significant difference in RFS between patients who underwent a segmentectomy vs. a lobectomy (five-year: 81.3% vs. 68.2%, *p* = 0.225) [59]. An additional retrospective study of 555 stage IA NSCLC patients by Ikeda et al. examining RFS and OS between wedge resection, segmentectomy, and lobectomy demonstrated that within STAS+ patients, both RFS (five-year: 70.6% vs. 72.6%) and OS (five-year: 92.9% vs. 83.6%) were similar between patients who received segmentectomies or lobectomies; however, both RFS (five year: 19.3% vs. 72.6%, *p* < 0.001) and OS (five year: 41.3% vs. 83.6%, *p* = 0.001) were significantly worse in patients who received wedge resections vs. lobectomies [60]. In STAS+ patients, undergoing a wedge resection was an independent prognostic factor for worse OS and RFS [60]. Similarly, in stage I (≤2 cm) NSCLC disease, Yang et al. investigated RFS for lobectomy vs. segmentectomy vs. wedge resection which revealed significantly worse RFS for wedge vs. lobectomy (*p* = 0.024), but not between segmentectomy and lobectomy [51].

On the contrary, it warrants discussion that a minority of retrospective studies have demonstrated no difference in outcomes for STAS+ patients following sublobar resection vs. lobar resection. For example, a retrospective study of 567 stage I (≤2 cm) STAS+ tumors by Pan et al. demonstrated that patients undergoing sublobar resection had similar five-year OS (78.7% vs. 81.6%, HR: 0.976, 95% CI: 0.693–1.375, *p* = 0.890) and RFS (69% vs. 71.8%, HR: 1.017, 95% CI: 0.752–1.376, *p* = 0.914) compared to lobar resection patients [61]. Moreover, a subgroup analysis of patients aged 70 or older demonstrated superior five-year OS for sublobar patients compared to lobar patients (73.1% vs. 67.2%, HR: 0.636, 95% CI: 0.404–0.969, *p* = 0.040) [61]. Furthermore, a recent retrospective study of 3714 STAS+ clinical stage IA1-2 NSCLC propensity-scored matched patients by Pan et al. demonstrated similar outcomes between sublobar and lobar resections in clinical stage IA2 disease (five-year RFS: 85.8% vs. 88.8%, *p* = 0.253; five-year OS: 93.9% vs. 93.3%, *p* = 0.759) but worse outcomes for sublobar resections compared to lobar resections in clinical stage IA1 disease (five-year RFS: 61.8% vs. 76.0%, *p* < 0.001; five-year OS: 76.6% vs. 84.1%, *p* < 0.001) [57].

### 5.2. Importance of Surgical Margin Distance in STAS

An additional operative consideration is the surgical margin distance. Adequate tumor margin distance is important for the prevention of local recurrence [62]. With the growing adoption of sublobar resections for early-stage NSCLC and the growing evidence that sublobar resections have worse outcomes in STAS+ patients, tumor margin distance is an essential consideration in choice of resection extent. While the optimal margin distance is still debated, in sublobar resections, general recommendations are at least 2 cm, or the tumor size to reduce recurrence risk [63]. A recent narrative review by Nagano et al. recommended a margin > 1 cm or margin–tumor ratio (M/T) of ≥1 in sublobar resections of STAS early-stage NSCLC [64].

However, considering the nature of STAS, do these same general recommendations apply or does the margin need to be higher for STAS+ tumors? Studies examining this question have led to mixed results. In stage I (≤2 cm) STAS+ patients, Kadota et al. demonstrated that the median distance of STAS from the tumor edge was 1.5 mm with a 5 mm perimeter from the tumor capturing ~90% of all STAS cases [24]. In this cohort, the investigators noted that the maximum distance at which STAS was observed from the tumor edge was 1.35 cm [24]. Furthermore, another study of stage I adenocarcinoma patients by Eguchi et al. showed that margin/tumor ratio did not impact the higher risk of locoregional recurrence among STAS+ patients who completed a sublobar resection [50]. Ren et al. demonstrated the presence of STAS in 9% of residual lung segments from simulated sublobar resections in 100 lobectomy cases, more likely with a tumor–margin distance of ≤1.5 cm (*p* = 0.010) [53].

Despite the small number of studies and their limitations, the results suggest that margins in STAS+ tumors do not need to be different than the traditional recommendations for STAS- disease. Therefore, using the current available data, in STAS+ disease, surgeons should tailor their resection to guarantee a minimum surgical margin of 2 cm, but further investigation is required to more precisely define margin cutoffs.

### 5.3. Implications of STAS on Extent of Resection

Within the limitations of retrospective studies in Table 1, the majority support a more extensive anatomical resection for STAS+ tumors given the worse prognosis following sublobar resection in STAS+ disease. To date, the strongest evidence from retrospective studies supports a lobectomy for STAS+ disease. This is particularly important in the era of sublobar (wedge or segmentectomy) for early-stage NSCLC. In particular, a meta-analysis of 11 studies (5097 patients) in stage I lung adenocarcinoma demonstrated worse OS and RFS within STAS+ patients that underwent a sublobar resection compared to a lobectomy [58]. However, this meta-analysis did not distinguish between wedge resections and segmentectomies. The few retrospective studies which separate wedge resection and segmentectomy suggest that a segmentectomy may be non-inferior to a lobectomy in STAS+ disease [59,60]. Furthermore, as demonstrated by Pan et al. there may be a more nuanced role for sublobar resection in appropriately selected STAS+ patients [61]. It must be stressed that current clinical recommendations regarding extent of resection in STAS+ disease rely solely on retrospective studies. Prospective studies or RCTs are needed to delineate the nuances of this topic.

In summary, while STAS currently is a postoperative pathologic diagnosis, within current limitations, surgeons may opt to mitigate future risks by performing a lobectomy when the risk of STAS is increased based on patient and tumor characteristics and when tolerable to the patient. Furthermore, when electing to perform a sublobar resection, surgeons should attempt to ensure sufficient margins of at least 2 cm.

These results highlight the need for improved tools enabling preoperative STAS risk stratification and intraoperative detection as discussed in further detail below. This will ensure that surgeons are able to make the best-informed choice about which surgery is best for each patient.

## 6. Impact of STAS on Adjuvant Therapy

While adjuvant chemotherapy (ACT) is the standard of care for stage II+ NSCLC, ACT for stage IB disease remains controversial with inconsistent evidence [65,66]. The most recent NCCN NSCLC guidelines (v5.2026) do not routinely recommend ACT for stage IB disease but rather recommend it for stage IB patients with high-risk features such as poorly differentiated tumors, vascular invasion, wedge resection, VPI, and unknown lymph node status [67]. In contrast, current European Society for Medical Oncology (ESMO) and American Society of Clinical Oncology (ASCO) guidelines do not routinely recommend ACT for completely resected stage IB disease [68,69].

Retrospective studies have demonstrated a role for ACT in stage IB VPI+ disease, with significant improvements in OS and RFS, even in patients with smaller tumors (1–3 cm) [70,71]. With growing evidence that STAS is a high-risk NSCLC feature with poor prognostic implications akin to LVI and VPI, this poses the question if similar stage IB STAS+ patients would benefit from ACT as no current guidelines explicitly endorse ACT for stage IB STAS+ disease.

To date, the potential benefit of ACT for stage I STAS+ disease has been evaluated in several retrospective studies. In a retrospective study of approximately 3300 stage I adenocarcinoma patients, Chen et al. demonstrated improved OS (HR: 0.604, 95% CI: 0.397–0.919, *p* = 0.018) and DFS (HR = 0.565, 95% CI: 0.372–0.858, *p* = 0.007) in stage IB STAS+ patients that received ACT [72]. Conversely, for stage IA STAS+ disease, only patients who underwent a sublobar resection and then subsequently received ACT had improved OS (HR: 0.787, 95% CI: 0.359–0.949, *p* = 0.034) and DFS (HR: 0.703, 95% CI: 0.330–0904, *p* = 0.029) [72]. In a retrospective study of approximately 3300 stage I patients, Lv et al. demonstrated improved DFS in only stage IB STAS+ disease with high-risk features (i.e., poorly differentiated tumors, LVI, and VPI) but not stage IA STAS+ disease following ACT (*p* = 0.046) [73]. A retrospective propensity score matching (PSM) analysis of approximately 400 stage I patients again demonstrated worse OS in stage I STAS+ patients who did not receive ACT (HR = 1.675, 95% CI: 1.043–2.689, *p* = 0.033) [74]. Furthermore, a recent retrospective multi-center cohort PSM analysis with of 2619 patients demonstrated no significant improvement in OS or ACT in stage IA STAS+ lung adenocarcinoma patients [75]. Finally, a recent meta-analysis of five studies (2899 patients) examining ACT in stage I STAS+ disease, demonstrated significantly prolonged OS in stage I STAS+ patients who received ACT (HR: 0.61, 95% CI: 0.47–0.79, *p* < 0.001) and DFS (HR: 0.69, 95% CI: 0.48–0.99, *p* = 0.044) [76]. ACT had a significant survival benefit even in patients who had received a lobar resection (DFS: HR 0.61, 95% CI: 0.45–0.82, *p* = 0.001; OS: 0.60, 95% 0.42–0.55, *p* = 0.005) [76]. When stratified by stage IA vs. stage IB disease, the disease-free survival benefit was sustained for stage IB STAS+ disease (HR = 0.55, 95% CI 0.38–0.79, *p* = 0.001), but not stage IA disease (HR = 0.72, 95% CI: 0.42–1.25, *p* = 0.246) [76].

In summary, within the limitations of retrospective study, currently available data provide promising evidence supporting a role for ACT in STAS+ stage IB and a potential limited benefit in select STAS+ stage IA patients. However, further investigation—particularly randomized controlled trials—are needed to definitively demonstrate the potential benefit of ACT in stage I STAS+ patients. Until then, clinical uncertainty and a lack of consensus will remain.

## 7. Current Challenges and Limitations in STAS Detection

### 7.1. Preoperative Detection Challenges

While the development of an accurate non-invasive prediction tool for STAS would significantly improve surgical decision-making, the preoperative prediction of STAS remains challenging. While STAS cannot be directly visualized on computed tomography (CT), numerous studies have demonstrated associations between CT imaging tumor features and STAS.

Several retrospective studies have demonstrated that maximum tumor diameter is an independent predictor for the presence of STAS [77,78,79]. However, no tumor maximum diameter threshold has currently been determined to discriminate between STAS+ and STAS- disease with high fidelity. Furthermore, several retrospective studies have demonstrated associations between not only tumor size and but also percentage of the tumor solid component on imaging [77,78,80,81,82,83,84]. Based on these results, studies have suggested percentage of solid component thresholds for discriminating STAS+ vs. STAS−, with several studies recommending a consolidation-to-tumor ratio (CTR) of >0.5 [85,86,87]. Attempts to test the predictive efficacy of these CTRs in small sample sizes have demonstrated good sensitivity but only fair specificity [88,89]. In 276 lung adenocarcinoma patients, Kim et al. demonstrated that a 90% solid component on preoperative CT predicted STAS with 89.2% sensitivity and 60.3% specificity [88]. Similarly, in 190 stage IA adenocarcinoma patients, Qi et al. showed that an 83% CTR threshold predicted STAS with 91.5% sensitivity and 62.9% specificity [89]. While promising, there is no consensus on solid component threshold and the efficacy of CTRs in larger sample sizes remains unclear. Additional tumor characteristics that have been noted to be independent risk factors for STAS include presence of notching and lobulation [84,90].

**Figure 2:**
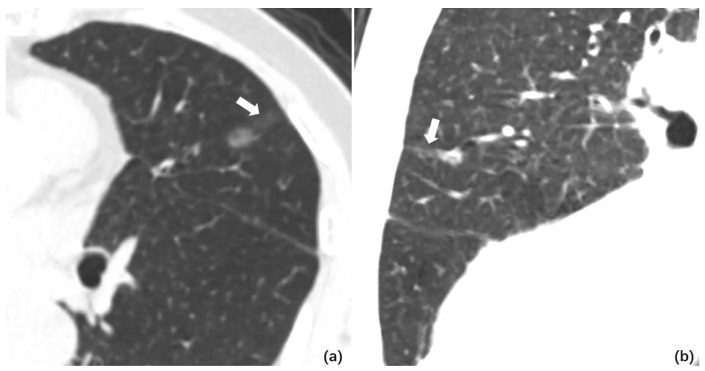
Example of Ground Glass Ribbon on CT Imaging. (**a**) Axial cut of preoperative CT imaging of a lung nodule (post-resection confirmed STAS+) with the ground glass ribbon sign: a nodule with blurred line (or “ribbon”) that arises from the nodule edge and extends to the surrounding lung (white arrow). (**b**) Multiplane reconstruction from same CT imaging from (**a**) with improved visualization of the ground glass ribbon sign (white arrow). Reproduced via Creative Commons CC BY license without alteration from: Qi L, Xue K, Cai Y et al. Predictors of CT Morphologic Features to Identify Spread Through Air Spaces Preoperatively in Small-Sized Lung Adenocarcinoma. *Font Oncol*. 2020;10:548430. Doi: 10.3389/fonc.2020.548430 [89].

Several retrospective studies have investigated potential associations between STAS, and other features noted on CT imaging including central low attenuation, air bronchogram, lobulation, pleural thickening, and vascular convergence [77,78,88]. Of these conventional CT features, lobulation demonstrated significant association with STAS in several retrospective studies [77,78,84]. More recently, a retrospective study of 190 stage IA adenocarcinomas by Qi et al. characterized a new CT imaging finding termed the “ground glass ribbon” (a ground-glass band with a blurred edge from the nodule, CT imaging example in Figure 2) which was significantly predictive of the presence of STAS [89]. This was subsequently confirmed in a prospective study of 336 stage IA NSCLC patients by Wang et al. [83].

Thus, while an accurate non-invasive prediction of STAS using CT imaging remains elusive, advancements within the field have identified numerous imaging features which are associated with STAS. Limitations with the predictive efficacy are likely partially attributable to the current lack of a comprehensive and precise definition of STAS. With advancements in artificial intelligence (AI), imaging radiomics and deep learning models have the potential to develop better predictive models. However, models will require validation in large-sample prospective studies.

### 7.2. Intraoperative Detection Challenges

Frozen section, an important tool for guiding the intraoperative treatment of pulmonary lesions due to its rapid tissue assessment, is a significant challenge for STAS. Current frozen section analysis is specific but not sensitive for STAS detection. Despite advancements, the accuracy of determining STAS remains poor with a sensitivity of 44–54% and specificity of 80–91% [19,20,50,91,92]. This has been hypothesized to be due to various factors including the size limitations of frozen sections (limited space beyond the tumor margin on frozen section) and parenchymal collapse on frozen section adjacent to the lesion. Studies examining STAS determination on frozen section in tumors ≤ 2 cm, reported limited sensitivity and specificity (sensitivity 55%, specificity 85%), but improved accuracy in small tumors with consolidation-to-tumor ratios (CTR) > 0.5 [92].

### 7.3. Lack of Universal Diagnostic Criteria and Moderate Interobserver Variability

Another significant challenge in the diagnosis of STAS is the lack of universal and precise criteria for STAS. This lack of universal and precise criteria has led to noted interobserver and intraobserver variability, particularly between distinguishing true STAS from artifacts resulting from tumor processing. There have been significant attempts to more precisely define STAS-including criteria based on distance to tumor edge and/or the number of tumor clusters [29,30,31,47,48,93]. A study by Villalba et al. concerning the accuracy and reproducibility of STAS detection demonstrated only moderate interobserver agreement (IOA) for STAS detection [20]. A panel of five thoracic pathologists reviewing 100 stage I lung adenocarcinoma frozen sections in two rounds with a consensus conference between rounds demonstrated a moderate initial IOA (κround-1: 0.453) [20]. This IOA only minimally improved following the consensus conference (κround-2: 0.506) [20]. Intraobserver agreement (ITA) was moderate and remained unchanged. The only significant predictor of diagnostic discordance was artifact presence [20]. These results demonstrate need for more precise standardized criteria or training for determining STAS (and differentiating it from artifact) to ensure global implementation and minimize interobserver variability.

### 7.4. Implications for Real-Time Operative Decision-Making

Considering the negative prognostic implications of STAS+ disease and potential benefit of a lobectomy, diagnosing STAS preoperatively or intraoperatively is ideal. However, as detailed above, challenges in preoperative imaging assessment, frozen section analysis, and interobserver variability, currently limit the ability to detect STAS with high confidence until final surgical pathology. Thus, as sublobar resections become increasingly performed, surgeons are currently limited in their operative decision-making regarding resection extent based on STAS.

## 8. Future Directions

### 8.1. Innovations in STAS Predictors and Detection

Despite the current challenges in predicting, diagnosing, and treating STAS+ NSCLC, advancements in technology and molecular associations are promising. These possess the opportunity to significantly improve STAS prediction and detection models through various techniques including radiomics and deep learning methods.

#### 8.1.1. Molecular Predictors of STAS

Molecular predictors of STAS represent an exciting future avenue in preoperative STAS risk stratification. This is particularly true considering advancements in molecular understandings of STAS and improvements in biomarker detection. Retrospective studies have noted several molecular associations with STAS including BRAF mutations, ALK rearrangements, ROS1 rearrangements, KRAS mutations, and negative PD-L1 status [28,39,48,52,94,95]. These potential molecular associations expand the potential data that can be used to develop preoperative risk-stratification STAS tools. However, these molecular associations require additional investigation to achieve consensus on inclusion in new and existing risk-stratification models.

#### 8.1.2. Application of Radiomics in STAS Prediction

Radiomics, the high-throughput extraction of large amounts of information from radiographic images, has shown significant promise in developing models. Imaging radiomic models have been developed from extracted tumor region of interest (ROI) radiomic features [85,96,97,98]. These models have exhibited AUC (area under the curve) between 0.63 and 0.802 in retrospective and prospective studies [85,96,97,98]. As STAS is present at the tumor margin, radiomic imaging models that extract peritumoral features (at various distances from the tumor margin) result in AUCs exceeding 0.8, performing better than radiomic predictive models relying solely on tumor features [99,100,101]. Furthermore, when radiomic imaging models are combined with clinical predictors of STAS, predictive efficacy improves, with Zhuo et al. demonstrating a combined radiomics and clinical signature models with AUC of 0.99 [101]. Overall, these models demonstrate a strong predictive ability, and with further refinement and reliance on artificial intelligence could be seamlessly and economically included into clinical algorithms.

#### 8.1.3. Application of Deep Learning Methods and Other AI Technology

Deep learning methods have been applied to both STAS prediction and detection, advancing the efficacy of both. For STAS prediction models, deep learning methods have led to improved predictive models compared to radiomics alone [102,103,104,105]. Furthermore, models that combine deep learning methods with radiomic features further outperform such as the combined model devised by Jin et al. which had a predictive AUC 0.94 on internal and AUC of 0.84 and 0.84 on two external validation cohorts [104]. Similarly, STAS predictive models based on deep learning methods combined with other AI techniques yield high predictive efficacy. For example, the model developed by Wang et al., which combined attention mechanisms with deep learning methods, resulted in a predictive AUC of 0.933 in the training cohort, 0.783 in the validation cohort, and 0.806 in the test cohort, outperforming a predictive model based on deep learning method alone [105].

Deep learning methods have also been applied to STAS detection with similar promise [106,107]. Feng et al. developed an AI-based deep learning STAS detection model (STASNet) in stage I lung adenocarcinoma patients which exhibited an AUC of 0.93 for detecting STAS at the tiles level and an AUC of 0.72–0.78 for detecting STAS at the while slide image level [107]. Additionally, introduction into the clinical environment subsequently improved STAS detection rate and identified three examples of occult STAS that were easily misidentified [107]. Moreover, a semi-quantitative score derived from STASNet and spatial location information (T10S) significantly correlated to DFS (HR: 3.517, 95% CI: 1.576–7.849, *p* = 0.002), enabling the risk stratification of STAS+ patients which is fundamental to personalized treatment for NSCLC patients [107].

Overall, AI-based technology has the potential to allow clinicians to make more informed, standardized preoperative appraisals of STAS risk, automating detection of STAS, decreasing variability in diagnosis, and advancing precision NSCLC treatment. However, this potential requires universal and precise guidelines for the classification of STAS.

## 9. Conclusions

In conclusion, since first being classified over a decade ago, investigations into STAS have increasingly demonstrated that it is a treatment-defining factor which should, in addition to other tumor and patient characteristics, influence NSCLC treatment strategy—particularly the extent of initial upfront surgical resection. While not universal, a majority of the current studies suggest that patients with tumor STAS should undergo a lobectomy, if they are candidates, despite recent RCTs demonstrating the potential benefits of sublobar resections amongst early-stage NSCLC tumors. Importantly, a major limitation of the current evidence surrounding STAS and optimal extent of surgical resection, is that existing data is derived from retrospective studies. As such, the choice of optimum resection extent is likely more nuanced. As demonstrated by retrospective studies directly comparing STAS+ patients undergoing segmentectomies vs. lobectomies, a segmentectomy may be non-inferior to a lobectomy in STAS+ disease [59,60]. Furthermore, some studies suggest a sublobar resection (segmentectomy or wedge resection) may be beneficial in STAS+ disease in select situations such as early-stage disease in patients aged 70 or older [61]. Thus, prospective trials are needed to delineate the nuances of resection extent in STAS+ NSCLC to distinguish if there is a particular STAS+ patient population in which a sublobar resection, particularly a wedge resection, is beneficial. To this end, future studies distinguishing between wedge resection and segmentectomy within sublobar resections are needed.

Furthermore, current limitations regarding STAS preoperative prediction modeling and intraoperative diagnosis limit the ability of surgeons to have strong confidence about STAS status until the final surgical pathology. Thus, at present, patients may undergo sublobar resections only to be revealed to have tumor STAS, potentially having completed the suboptimal operation. In these situations, especially in early-stage disease which is stage IB+, adjuvant therapy is a promising treatment option. Based on current retrospective studies, in early-stage disease, a sublobar resection followed by adjuvant therapy is likely an acceptable treatment strategy, but randomized controlled trials are needed to further evaluate this, with particular comparison to STAS+ patients who undergo a lobectomy [72,73,74,75,76].

As detailed previously, radiomics and AI-based prediction and detection models show great promise and are rapidly advancing. In the near future, these models should be implemented in the preoperative and/or intraoperative settings to allow surgeons to risk-stratify STAS probability or confirm STAS status intraoperatively and thereby make the best-informed patient-centered decision about which operation to complete. These models could also potentially be used to predict STAS-related recurrence risk and thereby guide adjuvant treatment decisions. This is the future of precision, patient-centered NSCLC treatment.

## Figures and Tables

**Table 1:** Studies Evaluating NSCLC Stratified by STAS and Extent of Resection.

Study	Year	Sample Size	Histology	Tumor Stage	STAS Prevalence	Resection Comparison	Recurrence Risk	RFS	OS
Kadota et al. [24]	2015	411 (SR = 120; LR = 291)	ADC	I (≤2 cm)	38%	SR vs. LR	STAS associated with higher recurrence in SR (42.6% vs. 10.9%, *p* < 0.001) but not in LR	N/A	N/A
Shiono et al. [55]	2018	514 (SR = 185; LR = 329)	All NSCLC	I (≤2 cm)	20.2%	SR vs. LR	STAS independent risk factor within SRs, but not LRs	N/A	STAS independent prognostic factor within SRs, but not LRs
Kadota et al. [52]	2019	735 (SR = 114; LR = 376)	ADC	I–IV	34%	SR vs. LR	Stage I: STAS+ SR higher than STAS+ LR (5-yr RFP: 48% vs. 66%, *p* = 0.010)	N/A	Stage I: STAS+ SR worse than STAS+ LR (5-yr: 43% vs. 77%, *p* < 0.001)
Eguchi et al. [50]	2019	1497 (SR = 527; LR = 970)	ADC	I	40.5%	SR vs. LR	STAS+ SR higher than STAS+ LR (SHR: 2.84, 95% CI: 1.59–5.08)	N/A	N/A
Ren et al. [53]	2019	752 (SR = 118; LR = 634)	All NSCLC	IA	28.7%	SR vs. LR	N/A	STAS independent risk factor for worse RFS within SRs, but not LRs (*p* < 0.001)	STAS independent risk factor for worse OS within SRs, but not LRs (*p* < 0.001)
Kagimoto et al. [59]	2021	609 (Segmentectomy = 107; LR = 186)	ADC	IA	48.1%	Segmentectomy vs. LR	N/A	STAS+ segmentectomy similar to STAS+ LR (81.3% vs. 68.2%, *p* = 0.225)	N/A
Yang et al. [58]	2021	5097	ADC	I	N/A	SR vs. LR	N/A	STAS+ SR worse than STAS+ LR	STAS+ SR worse than STAS+ LR
Ikeda et al. [60]	2023	555 (Wedge = 70; Segmentectomy = 68; LR = 417)	All NSCLC	IA	26.7%	Wedge vs. LR; Segmentectomy vs. LR	N/A	STAS+ segmentectomy similar to STAS+ LR; STAS+ wedge worse than STAS+ LR (5-yr: 19.3% vs. 72.6%, *p* < 0.001)	STAS+ segmentectomy similar to STAS+ LR; STAS+ wedge worse than STAS+ LR (5-yr: 41.3% vs. 83.6%, *p* = 0.001)
Chen et al. [54]	2024	212 (SR = 47, LR = 165)	ADC	I (≤2 cm)	43.9%	SR vs. LR	N/A	N/A	STAS+ SR worse than STAS+ LR (*p* = 0.007)
Axtell et al. [38]	2025	421 (SR = 103; LR = 318)	All NSCLC	I–IIIA	23%	SR vs. LR	Stage I (≤2 cm): STAS+ SR non-significantly higher than STAS+ LR	N/A	Stage I (≤2 cm): STAS+ worse within SR, but not within LR (73% vs. 95%)
Pan et al. [61]	2025	567 (SR = 179; LR = 388)	ADC	I (≤2 cm)	100%	SR vs. LR	N/A	STAS+ SR similar to STAS+ LR (HR: 1.017, 95% CI: 0.752–1.376)	STAS+ SR similar to STAS+ LR (HR 0.976, 95% CI: 0.693–1.375)
Yang et al. [51]	2025	445 (SR = 77; LR = 81)	NSCLC	I (≤2 cm)	35.5%	SR vs. LR	STAS+ SR higher than STAS+ LR (SHR: 7.60, 95% CI: 1.85–31.33)	STAS+ SR worse than STAS+ LR (66.1% vs. 88.8%, *p* = 0.042)	STAS+ SR similar to STAS+ LR (80.3% vs. 91.5%, *p* = 0.18)
Pan et al. [57]	2026	3714 (SR = 1238; LR = 2476)	NSCLC	IA1–2	100%	SR vs. LR	N/A	Stage IA1: STAS+ SR similar to STAS+ LR (5-yr: 85.8% vs. 85.8%, *p* = 0.253) Stage IA2: STAS+ SR worse than STAS+ LR (5-yr: 61.8% vs. 76.0%, *p* < 0.001)	Stage IA1: STAS+ SR similar to STAS+ LR (5-yr: 93.9% vs. 93.3%, *p* = 0.759) Stage IA2: STAS+ SR worse than STAS+ LR (5-yr: 76.6% vs. 84.1%, *p* < 0.001)

Abbreviations: ADC: adenocarcinoma; HR: hazard ratio; LR: lobar resection; NSCLC: non-small cell lung cancer; RFP: recurrence-free probability; SHR: subhazard ratio; SR: sublobar resection; STAS: spread through air spaces.

## Data Availability

No new data were created or analyzed in this study. Data sharing is not applicable to this article.

## Abbreviations

The following abbreviations are used in this manuscript:

ACT	Adjuvant chemotherapy
ADC	Adenocarcinoma
AI	Artificial intelligence
ASCO	American Society of Clinical Oncology
ESMO	European Society for Medical Oncology
IOA	Interobserver agreement
ITA	Intraobserver agreement
LR	Lobar resection
LVI	Lymphovascular invasion
NCCN	National Comprehensive Cancer Network
NSCLC	Non-small cell lung cancer
SR	Sublobar resection
STAS	Spread through air spaces
VPI	Visceral pleural invasion
WHO	World Health Organization
